# Orange peel ethanolic extract and physical exercise prevent testicular toxicity in streptozocin and high fat diet-induced type 2 diabetes rats via Nrf2/NF-kB signaling: In silico and in vivo studies

**DOI:** 10.1016/j.heliyon.2024.e39780

**Published:** 2024-10-24

**Authors:** Adeyemi Fatai Odetayo, Ayodeji Johnson Ajibare, Kazeem Bidemi Okesina, Tunmise Marryane Akhigbe, Ezekiel Abiola Olugbogi, Luqman Aribidesi Olayaki

**Affiliations:** a Department of Physiology, University of Ilorin, Ilorin, Nigeria; b Department of Physiology, Federal University of Health Sciences, Ila Orangun, Nigeria; c Department of Physiology, Lead City University, Ibadan, Nigeria; d Department of Medical Physiology, Faculty of Medicine and Pharmacy, University of Rwanda, Kigali, Rwanda; e Department of Agronomy, Osun State University, Osogbo, Nigeria; f Molecular Biology and Simulation Center, Ado-Ekiti, Nigeria

**Keywords:** Diabetes mellitus, Oxidative stress, Molecular docking, Apoptosis, Testicular dysfunction, Male fertility

## Abstract

**Background:**

Type 2 diabetes mellitus (T2DM) is a significant health issue affecting the quality of life including male reproductive functions. Orange peel ethanolic extract (OPEE) has been established to have antioxidant properties and has been shown to alleviate diabetic complications. This study determined to establish OPEE effect and physical exercise (EX) in T2DM-induced testicular dysfunction**.**

**Materials and methods:**

Thirty male Wistar rats were randomly distributed in five groups as follows: control group (received 1 ml/b.w of normal saline), and groups 2–5 were induced with diabetes, with group 2 left untreated, group 3 received 600 mg/kg b.w OPEE, group 4 was subjected to EX while group 5 was treated with OPEE alongside EX.

**Results:**

OPEE + EX ameliorated T2DM-induced decrease in sperm motility, count, and morphology and increased testicular lactate dehydrogenase, alkaline phosphate, gamma-glutamyl transferase, and lactate. T2DM-induced disruption of gonadotropin-releasing hormone, luteinizing hormone, follicle-stimulating hormone and, testosterone was also mitigated by OPEE + EX. In addition, OPEE + EX blunted T2DM-induced increase in oxidative stress, inflammatory, and apoptotic markers and the accompanied decrease in testicular nuclear factor erythroid 2–related factor 2 (Nrf2) and increase in nuclear factor kappa B (NF-κB). Also**,** OPEE + EX reversed T2DM-induced testicular histology distortion.

**Conclusions:**

The outcome of this study revealed that the combination of OPEE and EX ameliorated T2DM-mediated testicular damage via Nrf2/NF-κB signaling.

## Graphical abstract

**Figure:**
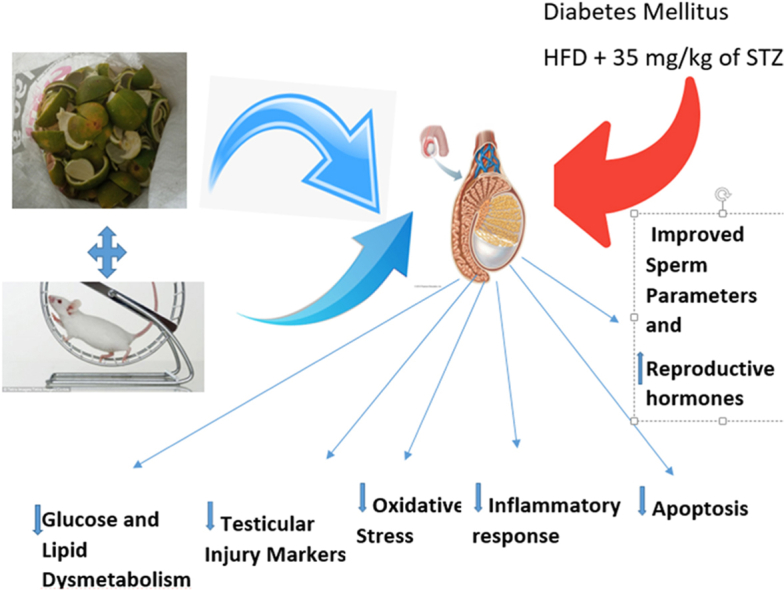

## 1 Introduction

Diabetes Mellitus (DM) is becoming more common, and there will likely be approximately 642 million diabetics worldwide in 2020 compared to 463 million in 2019 [1,2]. Chronic metabolic disease known as diabetes is typified by persistently high blood sugar levels resulting from either impaired secretion of insulin, its effect, or both [3]. Although DM pathogenesis has a complicated and multifaceted underlying cause, several studies has identified oxidative stress as a main player, and it has a major role in testicular function [4,5].

Oxidative stress occurs as a result of excessive generation of reaction oxygen species (ROS), which damage cellular constituents like proteins, lipids, and DNA when they build up in testes of patients suffering from type 2 diabetes mellitus (T2DM). Additionally, mechanistic research showed that long-term hyperglycemia causes NF-kB to become activated and consequently, the inhibition of Nrf2 [6]. This results in inflammatory response and a concurrent suppression of antioxidant activities [7]. When combined, these trigger apoptotic pathways that result in testicular cell death [8].

Conversely, orange peel extract has strong anti-inflammatory and antioxidant qualities and is abundant in flavonoids. Orange peel has been used traditionally in Persian medicine as a hepatoprotective agent [9], most likely because of its antioxidant qualities [10]. Additionally, it has been used to treat inflammatory disorders and dyspepsia in traditional Chinese medicine [11]. Although, investigations on animals have demonstrated the beneficial effects of orange peel [[12], [13], [14]], its role in T2DM-induced testicular injury has not been investigated. Also, exercise (EX) has been established as an underused lifestyle modification to lessen the toxic effects of T2DM [7]. Hence, orange peel and EX alone or in combination could ameliorate T2DM-induced testicular toxicity.

The regulation of Nrf2/NF-κB signaling may be the mechanism mediating these activities. OPEE increases the expression of antioxidant enzymes by activating Nrf2 [15]. EX has been proven in some studies to reduce NF-κB activation and play anti-inflammatory roles [16,17]. However, another study found that acute or intensive exercise may not affect NF-κB signaling [17], underscoring the need for more research. Therefore, we studied the combined effects of OPEE and EX on oxidative stress, inflammation, and testicular cell death in order to enhance testicular function and fertilization rate of male diabetic animals. We did this using both in-silico and in-vivo methods.

## 2 Materials and Methods

The thirty male Wistar rats utilized in this investigation were obtained from the University of Ilorin, and were handled as previously described [7,13,18]. “Briefly, the animals were given total access to food and water under a standard condition. The National Institutes of Health (NIH) procedures for Laboratory Animal Care were closely followed in the cautious handling of the animals, and the ARRIVE procedures for reporting experimental results were adhered to. The University of Ilorin Ethical Review Committee granted ethical approval (UERC/ASN/2017/1066) for the experimental protocol, which followed the National Research Council's criteria for the Care and Use of Laboratory Animals. Following a period of two weeks for acclimatization, the animals were randomly separated into five groups: control (treated with normal saline), diabetic untreated rat (DM-U), diabetic rats treated with 600 mg/kg b.w., diabetic rats treated with OPEE (DM + OPEE), diabetic rats treated with EX (DM + EX), diabetic rats treated with 600 mg/kg b.w. OPEE and EX (DM + OPEE + EX). All the treatments were via oral route and lasted for 28 days”.

### 2.1 Orange peel preparation

As previously described [13], “the oranges were obtained from the Lower Niger River Basin Citrus Farm in Ilorin, Kwara state and identified at the Department of Plant Biology, University of Ilorin with voucher No. UIH0001/159. The orange were carefully peeled, washed and air dried. After allowing the peels to air dry for four weeks, they were blended, and the resulting substance (about 500 g) was extracted using cold extraction and 95 % ethanol (4.5 L). Whatman filter paper 1 (Tokyo, Japan) was used to filter the peel extract, and an evaporator was used to remove the extraction solvent. After being dried and properly preserved, the extract was dissolved in 50 mg/mL of regular saline”.

### 2.2 Gas chromatography-mass spectrometry (GC-MS)

The analysis was performed at the Unilorin Chemical Engineering Laboratory. An HP Agilent Technologies (Agilent 19091S-433HP-5MS 5 % phenyl methyl siloxane) gas chromatograph fitted with a mass spectrometer was used to evaluate the ethanolic extract. CP-Sil 5 coating on HP-1 fused silica capillary column (30 m × 0.25 mm i.d.; film thickness, 0.25 μm). With a flow rate of 1 mL/min, helium served as the carrier gas. The injector and detector were both heated to 300 °C, and the injection was done in split mode (5:1). After being programmed to go from 20 to 250 °C at a rate of 4 °C per minute, the oven was kept at 35 °C for 5 min. The components were identified by comparing the fragmentation patterns with those documented in the literature, GC retention periods and retention index, and computer matching with the NIST11: library.

### 2.3 In silico

#### 2.3.1 Protein preparation

The following PDB IDs were used to simulate the target proteins' three-dimensional crystal structures: DPP-IV (3VJM) [19], Arpin (7JPN) [20], and LMPTP (7KH8) [21]. Hydrogen atoms were added to the target proteins (7KH8, 7JPN, and 3VJM) along with bond instructions [22], and the water molecules in five of the ligands were taken out of the structure of the protein-ligand complex [23]. Furthermore, side chains and loops that were absent from the primary tool were fixed. Further improvement of the proteins was achieved by generating tautomeric states at neutral pH and using the OPLS4 force field to restrict minimization for molecular docking.

### 2.4 Receptor grid generation

Receptor grid generation was used for protein-ligand docking, which identified the binding direction and size of the active site [23].

### 2.5 Ligand preparation

Thirty-seven orange peel bioactive compounds were obtained from published literature, and the NCBI PubChem database (https://pubchem.ncbi.nlm.nih.gov) provided the 2D structures of these orange peel bioactive compounds and the reference compounds. The Schrodinger suite's LigPrep function was then used in the OPLS4 forcefield to prepare the ligands. Only one stereoisomer per ligand was produced at a pH of 7.0 ± 2.0 when the compounds' ionization states were synthesized using the Epik module [24]. After co-crystallization, the ligands were produced in the same way as the other ligands and utilized as a drug reference.

### 2.6 Molecular docking

For the protein-ligand docking, Maestro 12.8 in the normal protocol of the Schrodinger package was used with the Glide tool [25]. Using the target proteins' crystal structures as a guide, a digital screening process was used to identify the compounds with the lowest docking scores among the produced proteins and chemicals created online.

### 2.7 Structure and interaction analysis

Discovery Studio 2020 Client was used to see the docked complexes once they were extracted as Mol2 files [26].

### 2.8 Exercise protocol

As previously described [13], “the animals were trained by letting them run for 5 min a day, two weeks before the commencement of the experiment (i.e., diabetes induction), on a modified exercise wheel at a speed of 20 m/min. The animals in the exercise groups continued to exercise for 28 days following the induction of type 2 diabetes. The pace of 20 m/min employed in this investigation is comparable to that of Luo et al. [27], and the exercise intensity is within the published intensity range for moderate exercise [28]”.

### 2.9 T2DM induction

Similar with previous findings [13], “the animals were randomized into two groups after they had been acclimated into the control and high-fat diet (HFD) groups. After the dietary adjustment was administered to the HFD rats for 12 weeks [29], an IP injection of 0.5 ml of a solution that contained 35 mg/kg of streptozocin (STZ) (Elabscience, Wuhan, China) dissolved in a citrate buffer (0.35–0.32) was administered to the rats [[30], [31], [32]]. Using a similar route, the control group was likewise given an equivalent volume of the vehicle (0.5 ml of citrate buffer).

While the control rats were fed the standard feed for 72 h after induction, the HFD (maize = 5.5 kg, wheat = 0.5 kg, ground nut cake = 5.5 kg, soya meal/cake/full fat = 12.5 kg, palm kernel cake = 5.0 kg, bone meal = 0.5 kg, methionine = 0.25, lysine = 0.25) was followed for four weeks. In this study, diabetic animals were classified as animals with greater than 300 mg/dl after 72 h. We chose this model because, although a modest dose of STZ targets the pancreatic β-cells to develop T2DM, it does not generate insulin resistance (IR), which is a defining feature of T2DM [30]. Animals fed the HFD acquired insulin resistance, in contrast to those given the STZ injection [33]. Therefore, research that gives animals a diet high in fat alongside a low dose injection of STZ to damage pancreatic β-cells to develop insulin resistance closely resembles the pathophysiology of type 2 diabetes”.

### 2.10 Sample collection

The rats were fasted overnight and 24 h after the treatment, they were sacrificed by the administration of 40 mg/kg of ketamine and 4 mg/kg of xylazine [34]. Using a cardiac puncture, blood samples were taken, and the serum was extracted for biochemical analysis by centrifuging the mixture for 5 min at 3000 rpm. To acquire the supernatant for biochemical examination, the adhering tissues to the left testes were removed, homogenized in an Eppendorf tube, and centrifuged for 10 min at 4 °C at 10,000 rpm. In addition, the right testis was taken out and histologically preserved using a bouin solution.

### 2.11 Sperm analysis

To test motility, a surgical blade was used to cut the cauda epididymis. The spermatozoa were then put on a sterilized glass slide, diluted with a 2.9 % sodium citrate dehydrate solution that had been warmed beforehand, and covered with a coverslip. A phase-contrast light microscope was used to see this [35,36]. 200 spermatozoa in total were counted in smears made using the Wells and Awa stains (0.2 g of Eosin and 0.6 g of Fast green diluted in distilled water) to examine the sperm count and morphology was determined as previously described [37,38].

### 2.12 Biochemical Assays

Using a digital glucometer, the glucose oxidase method was used to determine the terminal fasting blood sugar. Colorimetry and an ELISA kit were used to measure the lipid profile. As previously described [38], “the activities of testicular lactate dehydrogenase (LDH) and alkaline phosphatase (ALP) (Aggape Diagnostic, Switzerland), gamma-glutamyl transferase (GGT) (Pointe Scientific Inc., USA), and lactate concentration (Abcam, China) were measured. The concentration of gonadotropin-releasing hormone (Melsin, China) was determined according to the manufacturer's instructions. Likewise, testosterone, follicle-stimulating hormone (FSH), and luteinizing hormone (LH) were measured based on the manufacturer's guidelines (Bio-Inteco, UK).

Testicular malondialdehyde (MDA) was measured as previously reported [[39], [40]], and the methods of [41,42] were used to assess the levels of Oxidative stress markers such as; glutathione peroxidase (GPx), catalase (CAT), superoxide dismutase (SOD), and glutathione (GSH). The process for measuring malondialdehyde (a result of lipid peroxidation) and 2-thiobarbituric acid (TBA) for MDA involves evaluating the pink chromogen complex [(TBA) 2-malondialdehyde adduct] that forms when heated to an acidic pH. After treating the 200 μl sample with 500 μl of Trichloroacetic acid (TCA) to eliminate proteins, it was centrifuged for 10 min at 3000 rpm.

After that, 0.1 mL of the supernatant and 1 mL of 0.75 % TBA were combined, heated for 20 min at 100 °C in a water bath, and then chilled with ice water. Next, using a spectrophotometer set to 532 nm, sample/standard absorbance was measured and compared to a blank. An extrapolation from a standard curve was used to determine the concentration of TBARS.

For SOD, One milliliter of the sample and 9 mL of distilled water were used to create a 1:10 dilution. A reference cuvette containing 2.5 ml of buffer, 0.3 ml of substrate (adrenaline), and 0.2 ml of water was also used. A 10 ml flask with a flat bottom was filled with 4 ml of H2O2 solution (800 μmoles) and 5 ml of phosphate buffer; at 37 °C, a gentle swirl was used to incorporate 1 ml of the diluted enzyme preparation into the reaction mixture; at 60-s intervals, samples of the reaction mixture were taken out, and 1 ml of the sample was blown into 2 mL of dichromate/acetic acid reagent to measure the H2O2 content.

The quantity of catalase in the sample was determined by comparing its absorbance at 653 nm to a known catalase standard. An aliquot of the material was centrifuged for 5 min at 4000 rpm to deproteinize it for GSH. An equivalent volume of 4 % sulfosalicylic acid was added. Next, 0.5 ml of the supernatant was mixed with 4.5 ml of Ellman's reagent. A blank was made by mixing 4.5 ml of Ellman's reagent with 0.5 ml of the diluted precipitating agent. For GPx, the sample was incubated at 37 °C for 3 min to extract GPx. After adding 0.5 ml of 10 % trichloroacetic acid (TCA), the mixture was centrifuged at 3000 rpm for 5 min. The absorbance was measured at 412 nm using a blank as a reference after adding 1 ml of 5′-5′-dithiobis-(2-dinitrobenzoic acid) (DTNB) solution and 2 ml of phosphate buffer to the supernatant. The GPx activity was determined by plotting a standard curve.

A colorimetry approach (Fortress Diagnostic kit, Switzerland) was also used to evaluate the total antioxidant capacity (TAC), and an ELISA method (Elabscience, USA) was used to quantify Nrf2. Testicular tumor necrotic factor-alpha (TNF-α), interleukin-6 (IL-6), caspase 3, NF-κB, and C-reactive protein (CRP) were all detected using ELISA kits (Elabscience, USA).

For testicular histopathology, the testis was washed with toluene, dehydrated using ethanol series, and fixed in a bouin solution. The testes were placed at room temperature, covered with paraffin wax, and kept overnight at 60 °C in an incubator. The testes were then cut into 5 μm thick paraffin slices and stained with hematoxylin and eosin (H&E)”.

### 2.13 Statistical analysis

This was done with the GraphPad PRISM 8 software using Tukey's post hoc test and a one-way analysis of variance (ANOVA). The result was presented as mean ± standard deviation, at a statistical significance of P less than 0.05.

## 3 Results

### 3.1 In silico

The results of the complexes' docking scores at the proteins' active sites are displayed in Table 1, Table 2, Table 3 as well as in supplemental Figures. The best complex for 7JPN was found to be adenosine-5′-triphosphate, which had a docking score of −10.737; for 7KH8 and 3FXJ, Naringin had docking scores of −7.802 and −8.1, respectively. Concurrently, vicenin-2, which has a −11.445 docking score, is the greatest fit for the target protein according to the 3VJM.

**Table 1:** The docking score results of ligands from OPEE compounds at the active site of arpin.

Entry Name (7JPN)	Pubchem ID	MW (g/mol)	DS (kcal/mol)
Adenosine-5′-triphosphate	5957	507.18	−10.737
Didymin	16760075	594.6	−9.495
Eriocitrin	83489	596.5	−9.243
Vicenin 2	442664	594.5	−8.968
Narirutin	442431	580.5	−8.349
Hesperidin	10621	610.6	−8.153
Neohesperidin	442439	610.6	−7.365
Naringin	442428	580.5	−6.815
Sinensetin	145659	372.4	−5.538
Tangeretin	68077	266.33	−4.127

**Table 2:** The docking score results of ligands from OPEE compounds at the active site of dipeptidyl peptidase-4.

Entry Name (3VJM)	Pubchem ID	MW (g/mol)	DS (kcal/mol)
Vicenin 2	442664	594.5	−11.445
Eriocitrin	83489	596.5	−10.708
Hesperidin	10621	610.6	−10.084
Didymin	16760075	594.6	−9.538
Naringin	442428	580.5	−8.905
Narirutin	442431	580.5	−8.214
Neohesperidin	442439	610.6	−6.3
Tangeretin	68077	372.4	−5.16
Citronellal	7794	154.25	−4.756
Nerol	643820	154.25	−4.474
**Alogliptin**	**11450633**	**339.4**	**−3.408**

**Table 3:** The docking score results of ligands from OPEE compounds at the active site of low molecular weight protein tyrosine phosphatase.

Entry Name (7KH8)	Pubchem ID	MW (g/mol)	DS (kcal/mol)
Naringin	442428	580.5	−7.802
Narirutin	442431	580.5	−7.698
Hesperidin	10621	610.6	−7.264
Didymin	16760075	594.6	−5.061
Neohesperidin	442439	610.6	−4.981
alpha-PHELLANDRENE	7460	136.23	−4.264
Eriocitrin	83489	596.5	−4.164
Perillaldehyde	16441	150.22	−4.159
**Dihydrochloride**	**9552079**	**464.3**	**−3.966**
D-Limonene	440917	136.23	−3.945
Limonene	22311	136.23	−3.945

### 3.2 Gas chromatography-mass spectrometry

The organic components of OPEE are displayed by GC-MS in Table 4. Hexadecanoic acid, ethyl ester, was determined to have the lowest percent composition (1.6 %), while 5-hydroxymethylfurfural had the greatest percent composition (48.3 %).

**Table 4:** Summary of Identified compounds and their Kovat Retention Index.

S/n	Compound	% Comp.	Kovat R.I. (DB-5)	MS Data
1	Furfural	3.6	830 (Adams. 1995)	54, 67, 96, 179, 281
2	4H-Pyran-4-one, 2,3-dihydro-3,5-dihydroxy-6-methyl-	4.4	1134 (Da Silva et al.*,* 1999)	55, 72, 101, 144, 355
3	Benzofuran, 2,3-dihydro-	4.1	1368 (El-Sayed et al.*,* 2005)	55, 65, 91, 120, 341
4	5-Hydroxymethylfurfural	48.3	1224 (Da Silva et al.*,* 1999)	53, 69, 97, 126, 207
5	2-Methoxy-4-vinylphenol	6.1	1313 (El-Sayed et al.*,* 2005)	51, 107, 135, 150, 331
6	n-Hexadecanoic acid	10.0	1984 (Priestap et al.*,* 2003)	60, 73, 129, 256, 405
7	Hexadecanoic acid, ethyl ester	1.6	1993 (Adams, 1995)	60, 73, 88, 157, 451
8	9,12-Octadecadienoic acid (Z,Z)	3.6	2173 (Alvesa et al.*,* 2005)	51, 81, 109, 280, 529
9	Linoleic acid ethyl ester	1.7	2155 (Blagojevic et al.*,* 2006)	55, 67, 109, 281, 599

## 4 In vivo

### 4.1 Co-administration of OPEE and EX ameliorates T2DM-induced glucose and lipid dysmetabolism

**Fig. 1:**
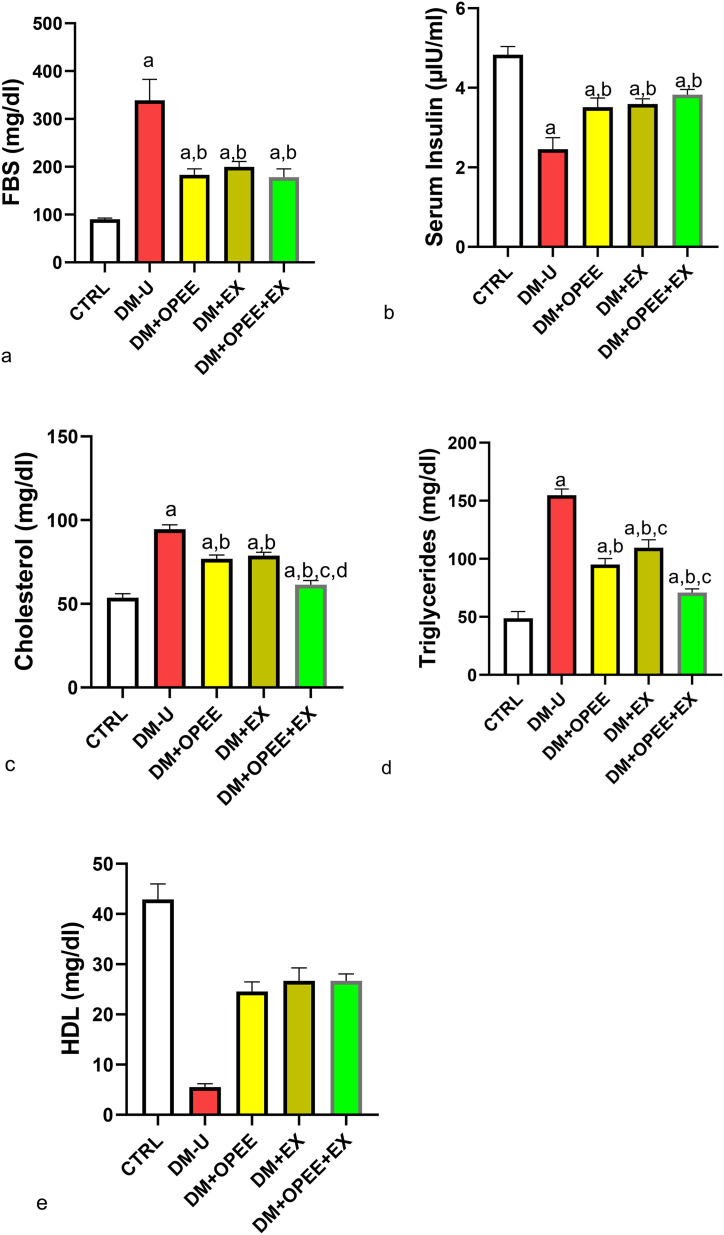
Effect of orange peel ethanolic extract and physical exercise on (a) FBS (b) serum insulin (c) serum cholesterol. ^a^P < 0.05 vs control, ^b^P < 0.05 vs DM-U, ^c^P < 0.05 vs DM + OPEE, ^d^P < 0.05 vs DM + Ex,. Data were analyzed by one way ANOVA and Tukey's posthoc test. CTRL: Control (normal saline); DM-U: Diabetic untreated DM + OPEE: Diabetic treated with orange peel extract; DM + EX: Diabetic, treated with exercise; DM + OPEE + EX: Diabetic treated with orange peel extract and exercise.

Fig. 1a–e illustrates how T2DM-induced increases in fasting blood sugar, TC, TG, and decreased serum insulin and HDL were lessened by OPEE and EX. The animals that received either OPEE or EX and their counterparts co-treated with OPEE + EX did not significantly differ in the ameliorative effect of OPEE and EX on T2DM-induced glucose dysmetabolism; however, a synergistic ameliorative effect of OPEE + EX was observed in T2DM-induced lipid dysmetabolism.

### 4.2 Co-administration of OPEE and EX ameliorates T2DM-impaired sperm quality

Table 5 demonstrated that T2DM-induced reductions in sperm motility, count, and normal morphology were ameliorated by OPEE and EX. It is noteworthy that although both OPEE and EX improved the low sperm quality, the rat that received both treatments showed a stronger improvement.

**Table 5:** Effect of orange peel ethanolic extract and physical exercise on sperm parameters.

	CTRL	DM-U	DM + OPEE	DM + EX	DM + OPEE + EX
					
**Sperm count (∗10^7)/ml**	5.88 ± 0.18	2.07 ± 0.01^a^	2.205 ± 0.24^a^^,^^b^	2.17 ± 0.14^a^^,^^b^	3.92 ± 0.21^a^^,b,c,^^d^
**Sperm Motility (%)**	76.23 ± 0.11	44.62 ± 0.23^a^	68.41 ± 0.31^a^^,^^b^	59.84 ± 0.26^a^^,^^b^^,^^c^	70.67 ± 0.13^a^^,b,c,^^d^
**Abnormal Sperm morphology (%)**	29.32 ± 0.53	67.84 ± 0.44^a^	32.43 ± 0.74^a^^,^^b^	31.89 ± 0.78^a^^,^^b^	29.10 ± 0.73^b^^,^^c^^,^^d^

a P < 0.05 vs control.

b P < 0.05 vs DM-U.

c P < 0.05 vs DM + OPEE.

d P < 0.05 vs DM + Ex,. Data were analyzed by one way ANOVA and Tukey's posthoc test.

### 4.3 Co-administration of OPEE and EX ameliorates T2DM-induced testicular injury and energy imbalance

**Fig. 2:**
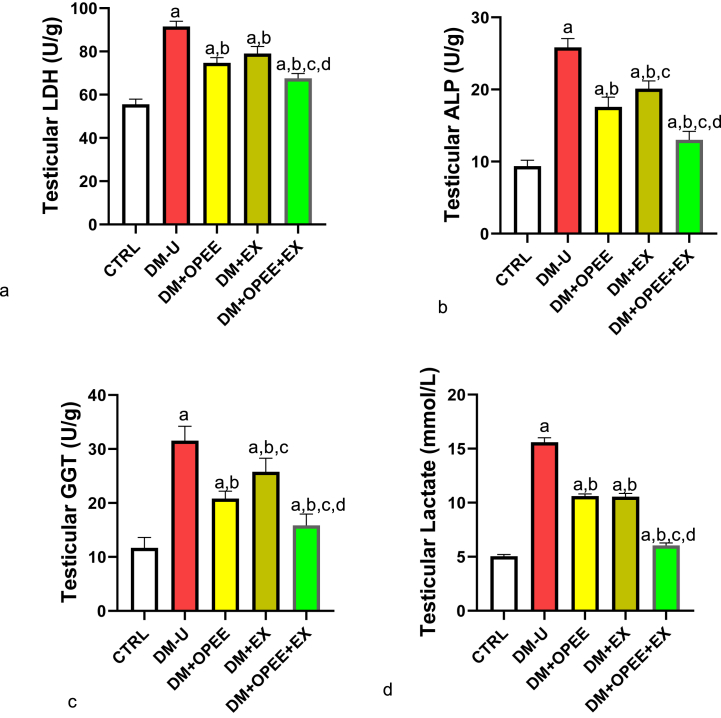
Effect of orange peel ethanolic extract and physical exercise on (a) LDH (b) ALP (c) GGT (d) lactate. ^a^P < 0.05 vs control, ^b^P < 0.05 vs DM-U, ^c^P < 0.05 vs DM + OPEE, ^d^P < 0.05 vs DM + Ex,. Data were analyzed by one way ANOVA and Tukey's posthoc test. CTRL: Control (normal saline); DM-U: Diabetic untreated DM + OPEE: Diabetic treated with orange peel extract; DM + EX: Diabetic, treated with exercise; DM + OPEE + EX: Diabetic treated with orange peel extract and exercise.

Fig. 2a–d illustrates how OPEE and EX considerably reduced the rise in testicular LDH, ALP, GGT, and lactate that was brought on by T2DM. Animals in the DM + OPEE + EX group recovered better from the T2DM-induced increase in testicular damage and energy imbalance, although animals treated with OPEE alone showed a better ameliorative effect than animals in the DM + EX group.

### 4.4 Co-administration of OPEE and EX ameliorates T2DM-induced hormonal imbalance

As shown in Table 6, T2DM significantly decreased serum GnRH, LH, FSH, and testosterone compared with the control, whereas OPEE and EX ameliorated these observed alterations compared to the DM-U. However, the alterations were more ameliorated in the animals that received OPEE and EX co-treatment when compared to those that received only OPEE or EX

**Table 6:** Effect of orange peel ethanolic extract and physical exercise on reproductive hormones.

	CTRL	DM-U	DM + OPEE	DM + EX	DM + OPEE + EX
**GnRH(mIU/mL)**	10.40 ± 0.4	5.03 ± 0.23^a^	8.02 ± 0.28^a^^,^^b^	8.12 ± 0.26^a^^,^^b^	9.90 ± 0.75^a^^,b,c,^^d^
**FSH (mIU/ml)**	4.79 ± 0.134	1.07 ± 0.011^a^	2.26 ± 0.24^a^^,^^b^	2.14 ± 0.139^a^^,^^b^	3.92 ± 0.21^a^^,b,c,^^d^
**LH (mIU/ml)**	6.14 ± 0.19	1.61 ± 0.57a	3.07 ± 0.35a,b	2.33 ± 0.22a,b,c	5.63 ± 0.38a,b,c,d
**Testosterone (ng/ml)**	2.76 ± 0.17	0.81 ± 0.13^a^	1.36 ± 0.26^a^^,^^b^	1.24 ± 0.113^a^^,^^b^^,^^c^	2.08 ± 0.11^a^^,b,c,^^d^
					
					

a P < 0.05 vs control.

b P < 0.05 vs DM-U.

c P < 0.05 vs DM + OPEE.

d P < 0.05 vs DM + Ex,. Data were analyzed by one way ANOVA and Tukey's posthoc test.

### 4.5 Co-administration of OPEE and EX ameliorates T2DM-induced oxidative stress

**Fig. 3:**
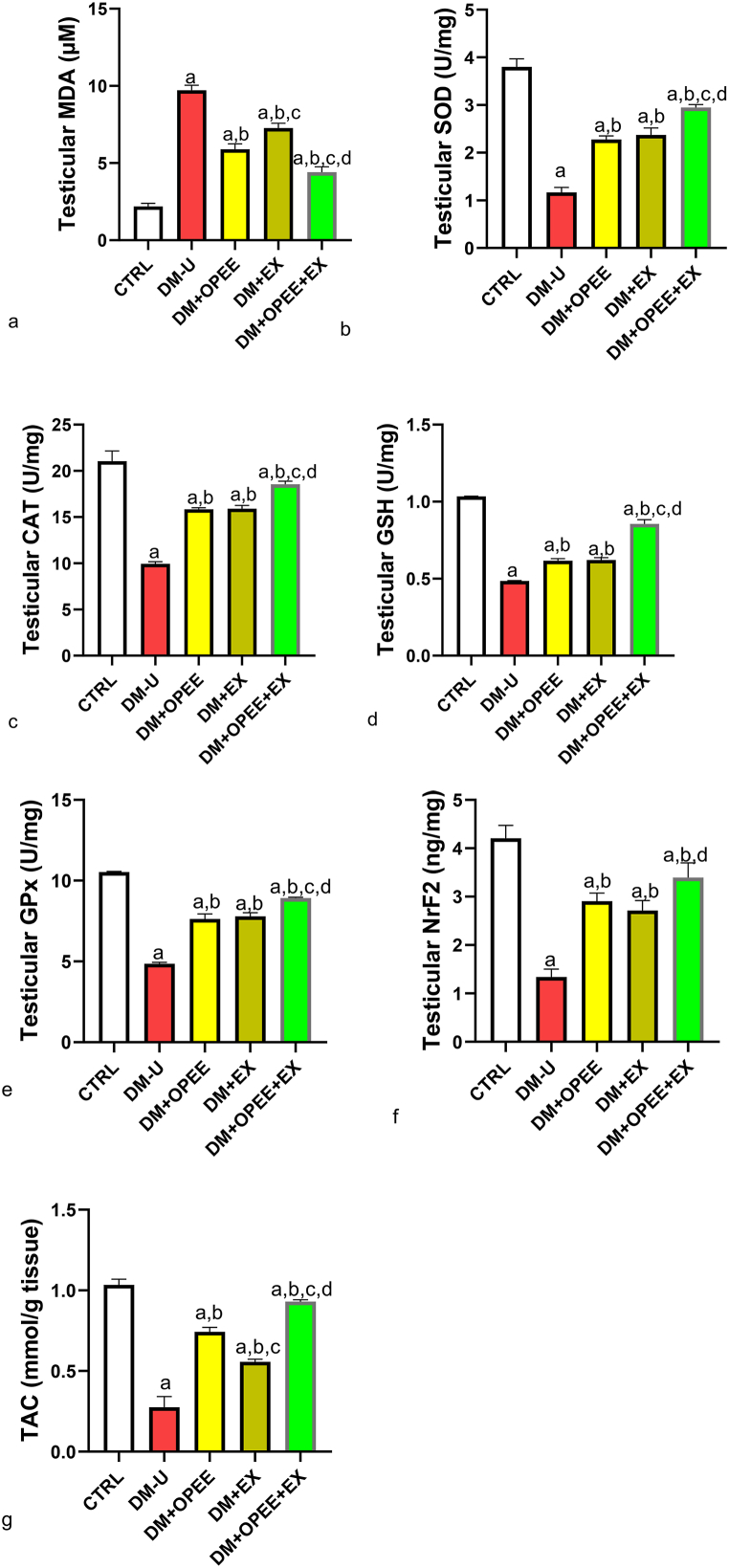
Effect of orange peel ethanolic extract and physical exercise on testicular (a) MDA (b) SOD (c) CAT (d) GSH (e) GPx (f) Nrf2 (g) TAC. ^a^P < 0.05 vs control, ^b^P < 0.05 vs DM-U, ^c^P < 0.05 vs DM + OPEE, ^d^P < 0.05 vs DM + Ex,. Data were analyzed by one way ANOVA and Tukey's posthoc test. CTRL: Control (normal saline); DM-U: Diabetic untreated DM + OPEE: Diabetic treated with orange peel extract; DM + EX: Diabetic, treated with exercise; DM + OPEE + EX: Diabetic treated with orange peel extract and exercise.

When comparing the testicular MDA of the animals in the DM-U group to those in the control group, it was shown that the increase in MDA caused by T2DM was attenuated by OPEE and EX. Rats given OPEE showed a discernible decrease in testicular MDA compared to rats in the exercise group. Additionally, compared to OPEE or EX treatment alone, a combination of OPEE and EX led to a further drop in MDA. Furthermore, in comparison to the control, OPEE and EX reversed T2DM-induced decrease in the activities of testicular SOD, CAT, GSH, GPx, and Nrf2. Once more, the animals receiving both OPEE and EX co-treatment had a more marked ameliorative impact on these antioxidant enzymes. Additionally, when comparing diabetic untreated rats to the control group, TAC was significantly lower; however, this drop was lessened in rats receiving either OPEE or EX treatment (Fig. 3a–g). It's interesting to note that rats receiving OPEE had a stronger ameliorative impact than rats receiving EX. Additionally, compared to rats treated with either OPEE or EX alone, there was a further rise in TAC in animals co-treated with both.

### 4.6 Co-administration of OPEE and EX ameliorates T2DM-induced inflammatory response

**Fig. 4:**
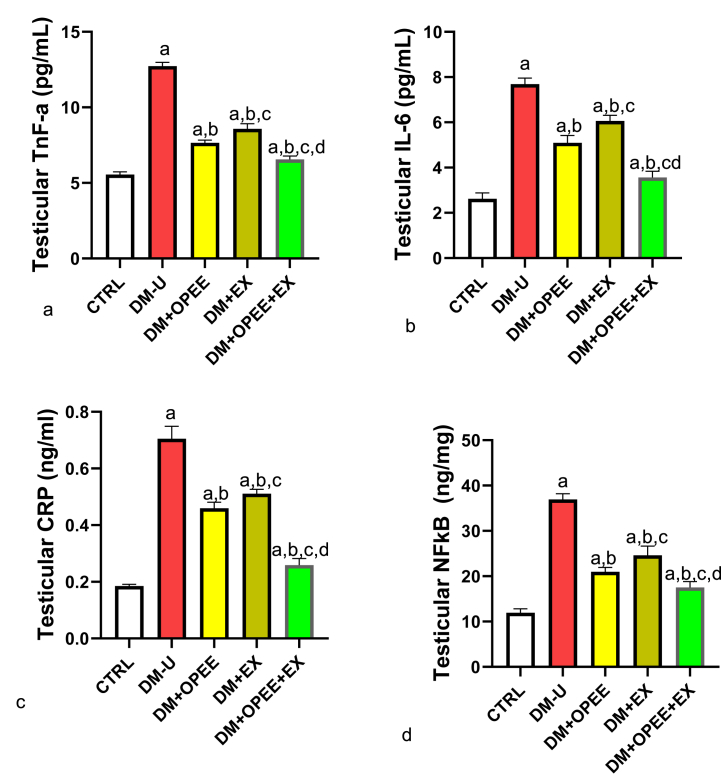
Effect of orange peel ethanolic extract and physical exercise on testicular (a) Tnf-α (b) IL-6 (c) CRP (d) NF-κB. ^a^P < 0.05 vs control, ^b^P < 0.05 vs DM-U, ^c^P < 0.05 vs DM + OPEE, ^d^P < 0.05 vs DM + Ex,. Data were analyzed by one way ANOVA and Tukey's posthoc test. CTRL: Control (normal saline); DM-U: Diabetic untreated DM + OPEE: Diabetic treated with orange peel extract; DM + EX: Diabetic, treated with exercise; DM + OPEE + EX: Diabetic treated with orange peel extract and exercise.

Testicular TNF-α, IL-6, CRP, and NF-κB levels were higher after T2DM therapy compared with the control (Fig. 4a–d). In contrast, DM + OPEE and DM + EX treatment decreased T2DM levels when compared to the DM-U group. Furthermore, animals treated with OPEE showed a greater reduction in inflammatory markers than mice given physical exercise. In addition, when compared to their counterparts in the DM + OPEE and DM + EX groups, animals in the DM + OPEE + EX group showed an even greater decrease in these inflammatory markers.

### 4.7 Co-administration of OPEE and EX ameliorates T2DM-induced apoptosis

**Fig. 5:**
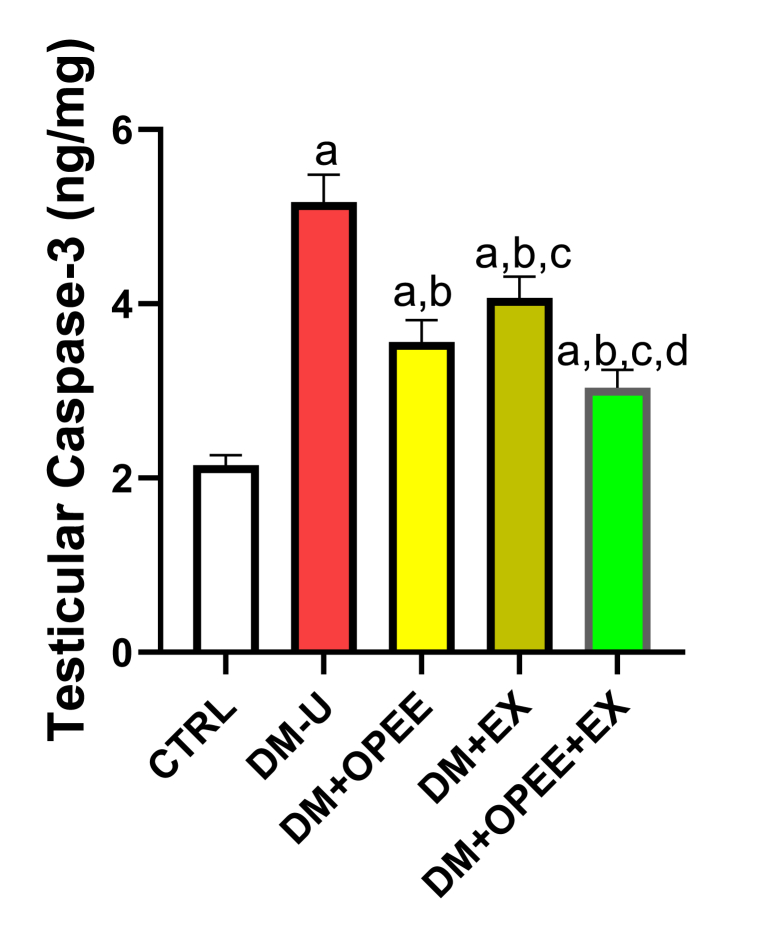
Effect of orange peel ethanolic extract and physical exercise on testicular caspase 3. ^a^P < 0.05 vs control, ^b^P < 0.05 vs DM-U, ^c^P < 0.05 vs DM + OPEE, ^d^P < 0.05 vs DM + Ex,. Data were analyzed by one way ANOVA and Tukey's posthoc test. CTRL: Control (normal saline); DM-U: Diabetic untreated DM + OPEE: Diabetic treated with orange peel extract; DM + EX: Diabetic, treated with exercise; DM + OPEE + EX: Diabetic treated with orange peel extract and exercise.

Rats with diabetes had considerably higher levels of testicular caspase 3 activity than the control group. Comparing the DM-U group to the DM + OPEE and DM + EX treatments, on the other hand, caspase 3 activity was significantly lower (Fig. 5). Treatment with both OPEE and EX further reduced the T2DM-induced rise in caspase 3, even though either treatment reduced the observed increase in caspase 3 more in OPEE treatment mice.

### 4.8 Co-administration of OPEE and EX ameliorates T2DM-induced testicular histology distortion

**Fig. 6:**
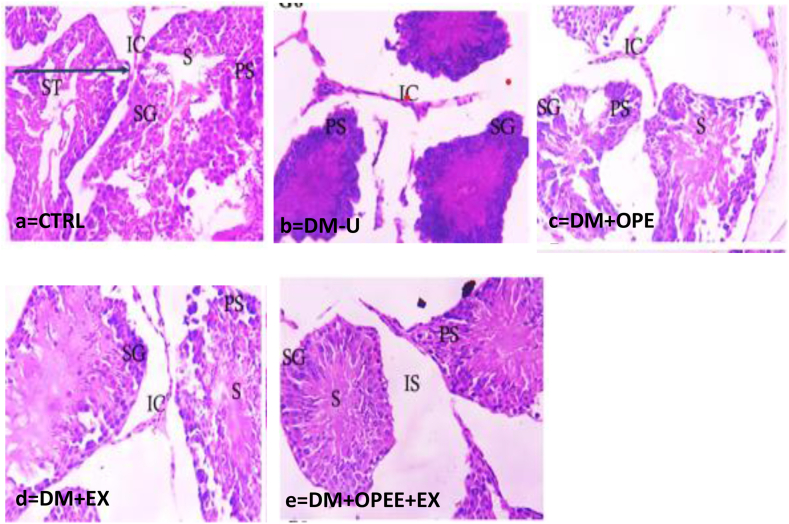
Histology of the testis. CTRL: Photomicrograph of control testis showing seminiferous tubules (ST) lined with series of spermatogenic cells; spermatogonia (SG), primary spermatocytes PS) and round (early) spermatids (S). Tubules are surrounded by basement membrane. The interstitial spaces in-between the tubules contains interstitial cell (IC). DM-U: shows complete loss of normal architecture of the seminiferous tubule (ST) with complete degeneration of the spermatogenic epithelial cells and corrugated basement membrane. DM + OPEE: shows irregular contour of the seminiferous tubule (ST), mild degeneration of spermatogenic (SG) epithelial cells and apoptotic cells. DM + EX: Alteration of seminiferous tubules (ST) and degeneration of spermatogenic cells, the basement membrane appear markedly disrupted. DM + OPEE + EX: Shows nearly preserved architecture of the seminiferous tubule with atrophy and empty Interstitial space (IS) Mg X 800. ST: Seminiferous tubule (Germinal epithelium), S: Spermatids, M: Myoid cells, IC: Interstitial cells, IS: Interstitial space, SG: Spermatogonium, PS: Primary spermatocytes, CTRL: Control, DM-U: Diabetes Mellitus Untreated, DM: Diabetes Mellitus, OPEE: Orange Peel Ethanolic Extract, EX: Exercise.

Rats in the DM-U group had markedly different testicular histology than the control group, as Fig. 6 illustrates, with the observed distortion being significantly reversed in the treatment groups. Comparing animals treated with combined therapy of OPEE and EX to those treated with either OPEE or EX, animals with the combination therapy revealed a greater ameliorative effect (Fig. 6a–e).

## 5 Discussion

This study shows that T2DM disrupted testicular functions in male Wistar rats, which is associated with impaired testicular enzymatic activities, energy imbalance, oxidative stress, and inflammation. These alterations were associated with Nrf2/NF-kB and caspase 3 signaling modulations. Also, this study revealed the therapeutic potential of OPEE and EX in T2DM rats. OPEE was considered in this study because the peel of sweet orange contains the majority of the health-promoting properties found in citrus fruits [43].

The ligands obtained from OPEE demonstrated notable binding affinity for the studied protein targets, as reflected by their docking scores in Table 1, Table 2, Table 3. Docking, a method in molecular modeling, predicts the optimal binding orientation of a ligand to its target protein, indicating a stable complex formation. Lower docking scores, signifying more negative binding energies, typically indicate stronger ligand-protein interactions.

In this investigation, reference ligands such as adenosine-5′-triphosphate, alogliptin, and dihydrochloride served as standards for assessing the binding affinity of the new compounds against arpin (7JPN), dipeptidyl peptidase-4 (3VJM), and protein tyrosine phosphatase (7KH8), respectively. Notably, sinensetin and tangeretin displayed comparatively lower docking scores against 7JPN, while the reference ligand achieved the highest score (−10.737 kcal/mol), indicating superior binding affinity. However, ligands from OPEE exhibited substantial binding energies, ranging from −9.495 to −6.815 kcal/mol, against 7JPN.

Vicenin 2 (−11.445 kcal/mol) emerged as the most promising compound against 3VJM, surpassing the reference ligand alogliptin (−3.408 kcal/mol). Strikingly, all compounds from OPEE showed higher binding energies against 3VJM, suggesting their potential as the enzyme's inhibitors. Similarly, at the active site of 7KH8, several ligands demonstrated favorable docking values indicative of strong affinity for the enzyme. Notably, naringin (−7.802 kcal/mol), narirutin (−7.698 kcal/mol), and hesperidin (−7.264 kcal/mol) exhibited higher docking scores compared to the reference ligand dihydrochloride (−3.966 kcal/mol).

Insulin Resistance (IR) is the defining characteristic of T2DM and is accompanied by dysmetabolism [44,45], which is a risk factor for T2DM complications. IR is defined as the inability of the cells in the muscles, adipose tissue and liver to truly respond to insulin and is unable to remove glucose from the blood resulting in carbohydrate and lipid dysmetabolism [46].The observed ameliorative effect of OPEE and EX on T2DM-induced glucose and lipid dysmetabolism is consistent with the findings from our molecular docking that OPEE inhibits the activities of DPP-4 and LMPTP. DPP 4, which is an important enzyme for maintaining body metabolism, has been shown to contribute to energy imbalance [47]. Also, LMPTP plays a negative role in insulin signaling and has been shown to increase insulin insensitivity [48,49]. Drugs that inhibit DPP-4 and LMPTP have been considered major drugs for preventing T2DM complications. Hence, OPEE can be considered an antidiabetic agent since it shares similar mechanisms of action with known antidiabetic drugs.

Data from this study showed that OPEE and EX prevented T2DM-impaired sperm quality by increasing sperm count, motility, and normal morphology. This result is in line with what was obtained from our molecular docking, which shows that OPEE inhibits Arpin, which is an inhibitor of the Arp2/3 complex. Arp2/3 complex maintains acrosomal reaction, capacitation, and numerous phases of spermatogenesis, such as acrosome biogenesis, flagellum formation, and nuclear processes, such as synaptonemal complex formation [50]. One important mechanism by which the Arp2/3 complex regulates spermatogenesis and sperm quality is by maintaining blood-testis-barrier (BTB). BTB allows nutrients from the systemic circulation to be available for the spermatogonial stem cells (SSCs), which are necessary for their proper differentiation during the seminiferous epithelial cycle of spermatogenesis [51]. Based on the established roles of the Arp2/3 complex, it is plausible that OPEE maintains spermatogenesis and sperm quality by maintaining BTB via its inhibitory effect on Arpin, which leads to the upregulation of the Arp2/3 complex activities.

The way in which OPEE and EX enhance spermatogenesis and sperm quality is through the maintenance of energy balance because spermatogenesis is an energy dependent process Testicular activities of ALP, LDH, GGT, and lactate are markers of energy balance, spermatogenesis, and Sertoli functions [52].The increase in the activities of ALP in the present study may have resulted from the release of this non-specific phosphate from the lysosomes of degenerating cells and the rapid catabolism of the testicular cells [53]. In addition, the energy used in spermatogenesis is received from, adenosine triphosphate (ATP), which comes from glycolysis and oxidative phosphorylation [54,55]. Since lactate is used as a preferred energetic substrate, it is synthesized by Sertoli cells and used by germ cells. The glucose synthesized in the Sertoli cells enters a cytosolic glycolysis where it is converted to lactate by LDH a process that provides main substrate for oxidative phosphorylation in the mitochondria [56,57]. The observed significant increase in testicular lactate after T2DM suggest energy disequilibrium [58] and may originate from the observed T2DM stimulated LDH enzyme activities which is a marker of testicular degeneration [40,59]. LDH plays a role of converting pyruvate to lactate during anaerobic state [60]. The significant increase in testicular LDH and lactate could be a compensatory adaptation to restore the observed T2DM-induced testicular damage. Furthermore, testicular GGT is a primary marker of Sertoli cell function, and its activities are directly related to the Sertoli cell's maturation and replication [40].The substantial rise in testicular GGT activities in T2DM animals suggests impaired Sertoli cell function and spermatogenesis [59]. In this regard, the co-treatment of OPEE and EX worked coordinately to reverse T2DM elevation in serum and testicular enzymes, ALP, LDH, GGT, and lactate, therefore signaling a return to physiological state from T2DM-induced energy imbalance, Sertoli cell damage and compromised spermatogenesis process.

The observed decline in GnRH, LH, FSH, and testosterone in the T2DM group aligns with previous findings [61,62]. The findings from this study that OPEE and EX synergistically ameliorated T2DM-induced hormonal imbalance suggested that OPEE and EX can maintain circulatory testosterone via the hypothalamic-pituitary-gonadal (HPG) axis. Physiologically, the hypothalamus maintains circulatory testosterone via the secretion and release of GnRH, which directly stimulates LH and FSH production from the pituitary. The LH produced from the pituitary gland is majorly responsible for stimulating testicular Leydig cells to produce testosterone, which, in turn, inhibits the pituitary and hypothalamus functions [63]. Hence, testosterone is regulated via hormonal-dependent mechanisms. OPE and EX can also improve circulatory testosterone by directly improving testicular activities. The testis is responsible for steroidogenesis (testosterone synthesis), and direct assault on the Leydig cells of the testis can impair steroidogenesis. The observed increase in cholesterol (precursor for testosterone) from this study could be a result of impaired activities of steroidogenic acute regulatory protein (StAR) that is responsible for transporting cholesterol for testosterone synthesis [64]. Hence, OPEE and EX synergistically increase circulatory and reproductive hormones by maintaining the HPG axis and testicular steroidogenic enzymatic activities.

Similar with our findings, it was earlier established that T2DM-induced gonadotoxicity is partly associated with the generation of ROS and free radicals [5,65]. OPEE possesses antioxidant properties with a free radical scavenging ability [66], while EX has also been established to reduce oxidative stress [67]. OPEE and EX may exert a gonado-protective effect in T2DM rats by improving the antioxidant defense system and suppressing oxidative stress. It is worth noting that although either OPEE or EX ameliorated T2DM-induced oxidative stress, their combination synergistically improved the antioxidant defense system.

In addition, the findings from this study that T2DM increased testicular TNF-α, IL-6, CRP, and NF-κB agreed with previous findings that reported similar findings in diabetic animals [68,69]. The observed T2DM-induced inflammatory response could result from its negative effect on testicular Nrf2. Nrf2 inhibits the activation of NF-κB signaling by improving the antioxidant status and heme oxygenase-1 (HO-1) expression [70], reducing ROS-mediated activation of NF-κB. Similarly, Nrf2 inhibits NF-κB signaling by preventing the degradation of nuclear factor of kappa light polypeptide gene enhancer in B-cells inhibitor, alpha (IκB-α), from preventing NF-κB mediated transcription [70]. Furthermore, overexpression of NF-κB can suppress the transcriptional activity of Nrf2, thereby leading to oxidative stress [71]. The ameliorative effect of OPEE and EX on T2DM-induced testicular toxicity could be via their modulatory effect on Nrf2/NF-κB signaling since OPEE and EX synergistically ameliorated T2DM-induced decrease in Nrf2 and increase in NF-κB.

The increased testicular oxidative stress and inflammatory response following T2DM explains the increase in apoptotic marker (caspase 3) observed in DM-U rats. Since oxidative stress can trigger inflammation and inflammation can also stimulate oxidative stress [72,73], T2DM possibly stimulates the intrinsic apoptotic pathway via oxido-inflammatory testicular damage [40]. Treatment with OPEE and EX reduced apoptosis in T2DM rats. The antiapoptotic activities of OPEE and EX in the testis of T2DM rats may be due to their antioxidant and anti-inflammatory properties in the testis since oxidative stress and inflammation form a vicious cycle with apoptosis [40].

The biological activities of OPEE extract against T2DM may be due to the presence of some bioactive constituent compounds. Orange peel extract contains flavonoids such as hesperidin and naringin and has been reported to have anti-inflammatory, antioxidant, and antiapoptotic activities [[74], [75], [76], [77]]. In fact, Nrf2/Nf-kb signaling has been established as the major mechanism of action for flavonoids [78]. Other components, such as tannins and saponins, have also been reported to have antioxidant activities. Furthermore, 5-Hydroxymethylfurfural acts as an antioxidant agent by preventing oxidative damage through its free radical scavenging activities and the upregulation of antioxidant enzymatic activities [79]. Also, n-hexadecanoic acid has been shown to have antioxidative and anti-inflammatory activities [80,81]. The anti-inflammatory activities and, subsequently, the antioxidative role of n-hexadecanoic acid might result from its inhibitory effect on Phospholipase A2, similar to the mechanism of action of other fatty acid derivatives that have been implicated in the management of inflammation [80].

## 6 Conclusion and future perspectives

To sum up, our study shows that via modifying the Nrf2/NF-κB signaling pathway, which is crucial for preserving redox homeostasis, OPEE and EX worked in concert to improve T2DM-induced testicular toxicity. As a result, OPEE and EX show intriguing therapeutic potential for usage as a successful substitute therapy for patients with type 2 diabetes. Nevertheless, since this study was done on animals, more research will be required to determine OPEE and EX's potential in clinical settings. Longitudinal studies are also advised to assess the sustainability of the noted gains and keep an eye out for any possible negative effects or side effects related to continued therapy. Additionally, it is important to look into the consequences of each of the OPEE's identified components separately.

## CRediT authorship contribution statement

**Adeyemi Fatai Odetayo:** Writing – review & editing, Writing – original draft, Visualization, Validation, Supervision, Software, Resources, Project administration, Methodology, Investigation, Funding acquisition, Formal analysis, Data curation, Conceptualization. **Ayodeji Johnson Ajibare:** Writing – review & editing, Project administration, Methodology, Investigation. **Kazeem Bidemi Okesina:** Writing – review & editing, Validation, Methodology, Investigation. **Tunmise Marryane Akhigbe:** Writing – review & editing, Methodology, Funding acquisition. **Ezekiel Abiola Olugbogi:** Writing – review & editing, Software, Methodology, Investigation. **Luqman Aribidesi Olayaki:** Writing – review & editing, Visualization, Validation, Supervision, Software, Project administration, Methodology, Investigation, Conceptualization.

## Declarations

### Informed consent

N/A.

## Data availability

Data will be made available on request.

## Research funding

The study did not receive funds from any organization/institution. This study was funded alone by the authors’ financial contributions.

## Declaration of competing interest

The authors declare that they have no known competing financial interests or personal relationships that could have appeared to influence the work reported in this paper.

## List of Abbreviations

T2DM: Type 2 diabetic mellitus
OPE: Orange peel extract
FBS: Fasting blood sugar
Nrf2: nuclear factor erythroid 2–related factor 2
NF-κB: Nuclear factor kappa B
LH: luteinizing hormone
FSH: follicle-stimulating hormone
CRP: c-reactive protein
TAC: Total antioxidant capacity
SOD: super oxide dismutase
CAT: catalase
