# Supplementary material for: Orange peel ethanolic extract and physical exercise prevent testicular toxicity in streptozocin and high fat diet-induced type 2 diabetes rats via Nrf2/NF-kB signaling: In silico and in vivo studies
Source: Heliyon. 2024 Oct 24;10(21):e39780. doi: 10.1016/j.heliyon.2024.e39780 (PMC11567124; doi:10.1016/j.heliyon.2024.e39780)
Supplement: Multimedia component 1 [file mmc1.docx]

Orange peel ethanolic extract and physical exercise prevent testicular toxicity in streptozocin and high fat diet-induced type 2 diabetes rats via Nrf2/NF-kB signaling: In silico and In vivo studies

Adeyemi Fatai Odetayo,^1,2^ Ayodeji Johnson Ajibare,^3^ Kazeem Bidemi Okesina^4^, Tunmise Marryane Akhigbe ^5^, Ezekiel Abiola Olugbogi,^6^ and Luqman Aribidesi Olayaki ^1^

^1^Department of Physiology, University of Ilorin, Ilorin, Nigeria.

^2^ Department of Physiology, Federal University of Health Sciences, Ila Orangun, Nigeria

^3^ Department of Physiology, Lead City University, Ibadan, Nigeria

^4^Department of Medical Physiology, Faculty of Medicine and Pharmacy, University of Rwanda, Kigali, Rwanda

^5^ Department of Agronomy, Osun State University, Osogbo, Nigeria

^6^Molecular Biology and Simulation Center, Ado-Ekiti, Nigeria

Corresponding: Odetayo Adeyemi Fatai

Email address: [adeyemiodetayo@gmail.com](mailto:adeyemiodetayo@gmail.com) or adeyemi.odetayo@fushi.edu.ng

Phone number: 07032766035


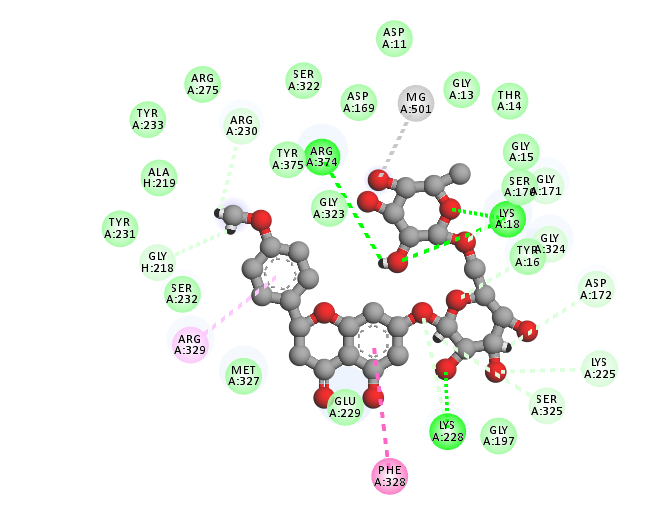

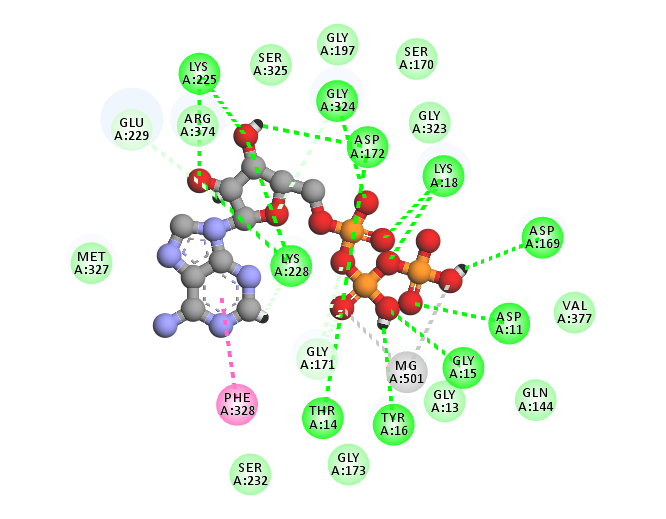


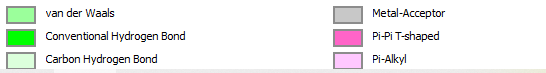


**Supplementary Figure 1**: The 2D structures of 7JPN_Didymin complex (left), and 7JPN_ Adenosine-5'-triphosphate complex (right) showing the interaction of amino acid residues and the compound at the target protein’s active site.

**Interactions**: 7JPN_Didymin

**van der Waals**: SER232, GLY218, TYR231, ALA219, TYR233, ARG275, ARG230, TYR375, GLY323, SER322, ASP169, ASP11, GLY13, THR14, GLY15, SER170, TYR16, GLY197, GLU229, and MET327. **Carbon hydrogen bond**: GLY171, GLY324, ASP172, LYS2255, and SER325. **Hydrogen bond**: ARG374, LYS18, and LYS228. **Pi-Pi T-shaped**: PHE328. **Pi-Alkyl**: ARG329. **Metal-Acceptor**: MG501.

**Interactions:** 7JPN_ Adenosine-5'-triphosphate

**van der Waals**: MET327, ARG374, SER325, GLY197, SER170, GLY323, VAL377, GLN144, GLY13, GLY173, and SER232. **Hydrogen bond**: LYS225, GLY324, ASP172, LYS18, LYS228, ASP169, ASP11, GLY15, TYR16, and THR14. **Carbon hydrogen bond**: GLU229, and GLY171. **Metal-Acceptor**: MG501. **Pi-Pi T-shaped**: PHE328.


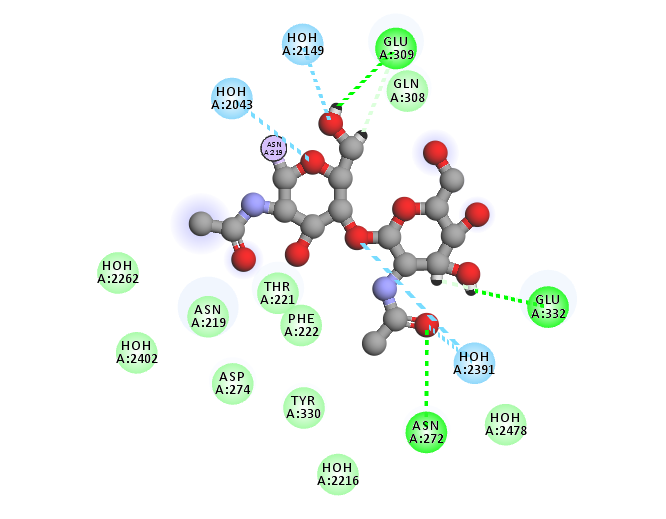

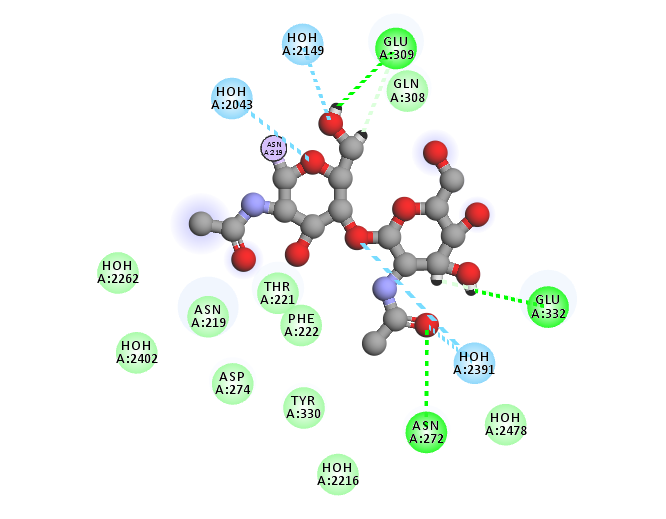


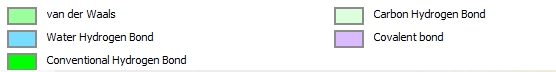


**Supplementary Figure 2**: The 2D structures of 3VJM_Vicenin2 complex (left), and 7JPN_ 3VJM_Alogliptin complex (right) showing the interaction of amino acid residues and the compound at the target protein’s active site.

**Interactions:** 3VJM_Vicenin2

**van der Waals**: GLN308, HOH2262, HOH2402, ASN219, THR221, PHE222, ASP274, TYR330, HOH2216. **Water hydrogen bond**: HOH2149, HOH2043, and HOH2391. **Hydrogen bond**: GLU309, GLU332, and ASN272. **Carbon hydrogen bond**: GLU309. **Covalent bond**: ASN219.

**Interactions:** 3VJM_Alogliptin

**van der Waals**: GLN308, HOH2262, HOH2402, ASN219, THR221, PHE222, ASP274, TYR330, HOH2216 and HOH2478. **Water hydrogen bond**: HOH2149, HOH2043, and HOH2391. **Hydrogen bond**: GLU309, GLU332, and ASN272. **Carbon hydrogen bond**: GLU309. **Covalent bond**: ASN219.


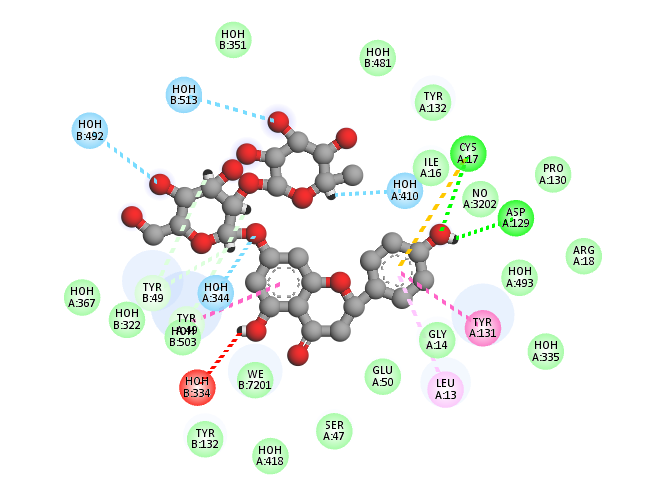

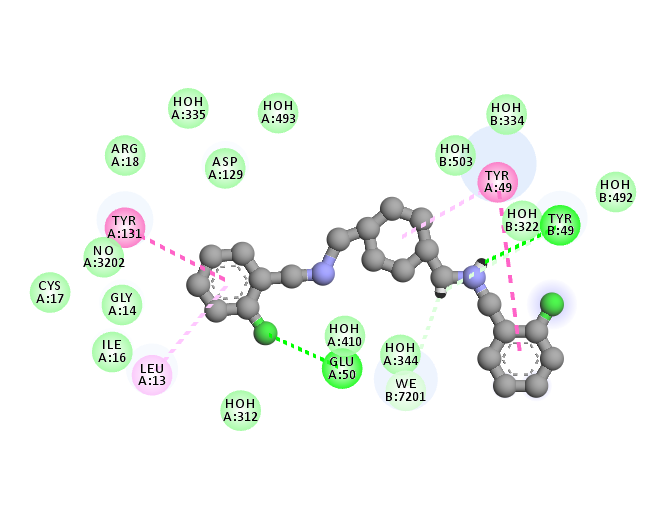


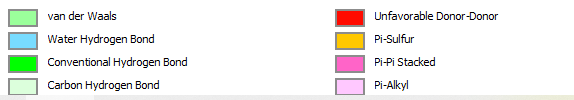


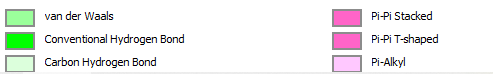


**Supplementary Figure 3**: The 2D structures of 7KH8_Naringin complex (left), and 7JPN_ 7KH8_Dihydrochloride complex (right) showing the interaction of amino acid residues and the compound at the target protein’s active site.

**Interactions:** 7KH8_Naringin

**van der Waals**: HOH351, HOH481, TYR132, ILE16, NO3202, PRO130, HOH493, ARG18, HOH335, GLY14, GLU50, SER47, HOH418, WE7201, TYR132, HOH503, HOH322, and HOH367. Water hydrogen bond: HOH513, HOH492, HOH410, and HOH344. Hydrogen bond: CYS17, and ASP129. Carbon hydrogen bond: TYR49, and TYR49. Unfavorable Donor-Donor: HOH334. Pi-Sulfur: CYS17. Pi-Pi Stacked: TYR131 and TYR49. Pi-Alkyl: LEU13.

**Interactions:** 7KH8_Dihydrochloride

**van der Waals**: HOH334, NO3202, ASP129, HOH492, ILE16, HOH322, ARG18, HOH335, GLY14, HOH410, HOH503, HOH344, and HOH312. **Hydrogen bond**: GLU50, and TYR49. **Carbon hydrogen bond**: WE7201. **Pi-Pi- Stacked and Pi-Pi T-Shaped**: TYR49, and TYR131. **Pi- Alkyl**: LEU13.


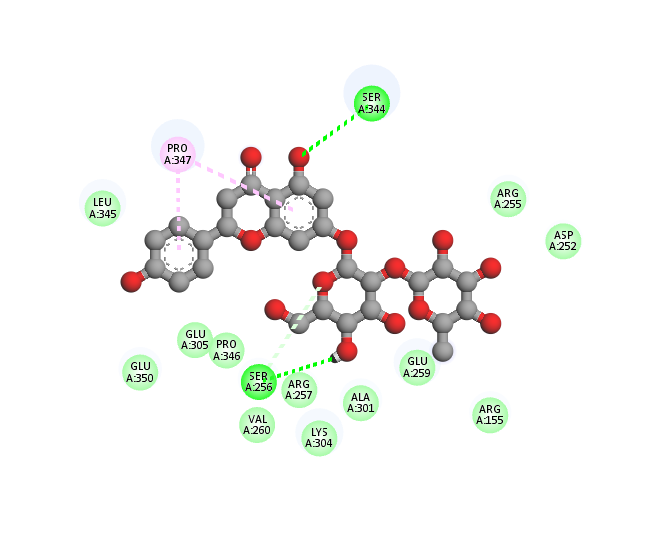

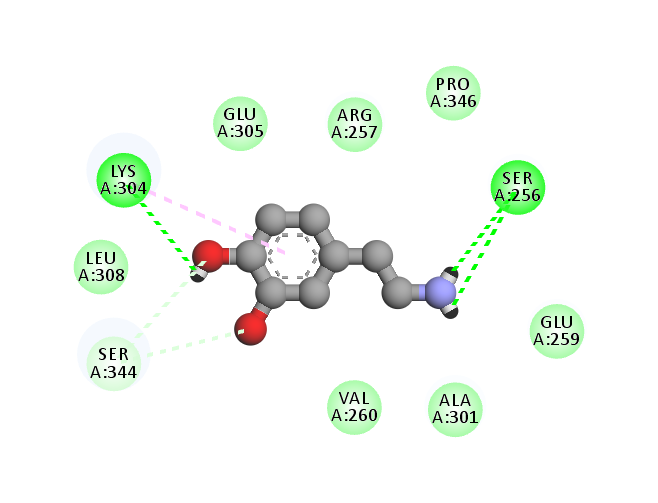


**
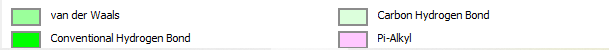
**

**Supplementary Figure 4**: The 2D structures of 3FXJ_Dopamine complex (left), and 3FXJ_ Naringin complex (right) showing the interaction of amino acid residues and the compound at the target protein’s active site.

**Interactions:** 3FXJ_Dopamine

**van der Waals:** GLU305, ARG257, PRO346, GLU259, ALA301, VAL260, and LEU308. **Hydrogen bond**: LYS304, and SER256. **Carbon hydrogen bond**: SER344. **Pi-Alkyl**: LYS304.

**Interactions:** 3FXJ_Naringin

**van der Waals**: ARG155, ASP252, ARG255, ARG257, GLU259, VAL260, ALA301, GLU305, PRO347, LEU345, and PRO346. Hydrogen bond: SER344, and SER256. Pi-Alkyl: PRO347. Carbon hydrogen bond: SER256.
